# Complete plastome sequence of *Nephelium topengii* (Merr.) H. S. Lo (Sapindaceae): an endemic species in Hainan

**DOI:** 10.1080/23802359.2020.1778556

**Published:** 2020-07-11

**Authors:** Zhi-Xin Zhu, Hong-Xin Wang, Hua-Feng Wang

**Affiliations:** Hainan Key Laboratory for Sustainable Utilization of Tropical Bioresources, College of Tropical Crops, Hainan University, Haikou, China

**Keywords:** *Nephelium topengii*, plastome, phylogeny, genome structure, Sapindaceae

## Abstract

*Nephelium topengii* is an evergreen tree of the Sapindaceae family, which can be used as timber. Here, we report and characterize the complete plastome of *N. topengii.* The complete plastome is 162,944 bp in length and contains the typical structure and gene content of angiosperm plastome, including two inverted repeat (IR) regions of 30,092 bp, a large single-copy (LSC) region of 85,909 bp and a small single-copy (SSC) region of 16,851bp. The plastome contains 130 genes, consisting of 80 unique protein-coding genes, 30 unique tRNA gene, 4 unique rRNA genes (5S rRNA, 4.5S rRNA, 23S rRNA and 16S rRNA). The overall A/T content in the plastome of *N. topengii* is 62.30%. The complete plastome sequence of *N. topengii* will provide a useful resource for the conservation genetics of this species as well as for phylogenetic studies in Sapindaceae.

## Introduction

*Nephelium topengii* (Merr.) H. S. Lo is a tropical plant in the Sapindaceae family which is an evergreen shrub or small tree ranging from 5 to 20 m tall (Xia and Paul 2007). It is only distributed in the low altitude forests of Hainan province (Yan et al. 2019). It is rich in tannins in peel and bark of *N. topengii.* At present, the complete plastome information and systematic position of *N. topengii* has not been reported. Hence, the genetic and genomic information is essential needed to aid to its resource exploitation and conservation. Here, we report and characterize the complete plastome of *N. topengii* (GenBank accession number: MT471264, this study) to benefit *N. topengii* germplasm collection, conservation and systematic studies.

In this study, *N. topengii* was sampled from Diaoluo mountain in Hainan province of China (109.87° E, 18.83° N). A voucher specimen (Wang et al., GPSII-001) and its DNA was deposited in the Herbarium of the Institute of Tropical Agriculture and Forestry (code of herbarium: HUTB), Hainan University, Haikou, China.

The experiment procedure is as reported in Zhu et al. (2018). Around six Gb clean data were assembled against the plastome of *Litchi chinensis* (KY635881.1) (Rivarola et al. 2011) using MITO bim v1.8 (Le-Petit-Quevilly, France) (Hahn et al. 2013). The plastome was annotated using Geneious R8.0.2 (Biomatters Ltd., Auckland, New Zealand) against the plastome of *Populus lasiocarpa* (KX641589.1). The annotation was corrected with DOGMA (Wyman et al. 2004).

The plastome of *N. topengii i*s found to possess a total length 162,944 bp with the typical quadripartite structure of angiosperms, contains two Inverted Repeats (IRs) of 30,092 bp, a Large Single-Copy (LSC) region of 85,909 bp and a Small Single-Copy (SSC) region of 16,851 bp. The plastome contains 130 genes, consisting of 80 unique protein-coding genes (seven of which are duplicated in the IR), 30 unique tRNA genes (seven of which are duplicated in the IR) and 4 unique rRNA genes (5S rRNA, 4.5S rRNA, 23S rRNA and 16S rRNA). The overall A/T content in the plastome of *N. topengii* is 62.30%, which the corresponding value of the LSC, SSC and IR region were 62.40%, 68.60% and 58.30%, respectively.

**Figure 1.**
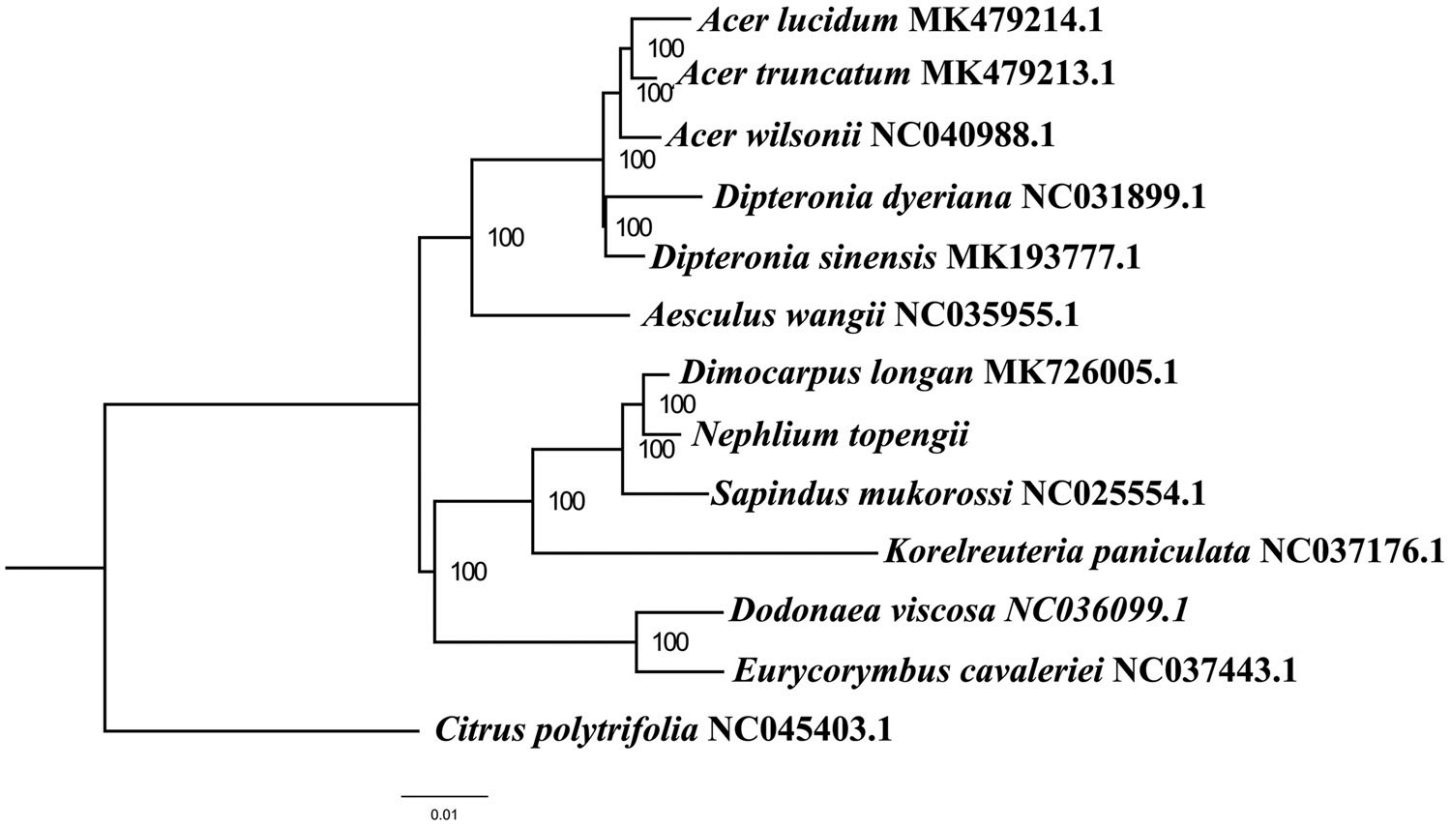
The best ML phylogeny recovered from 13 complete plastome sequences by RAxML. Accession numbers: *Nephelium topengii* (GenBank accession number, MT471264, this study), *Acer lucidum* MK479214.1, *Acer truncatum* MK479213.1, *Acer wilsonii* NC040988.1, *Dipteronia dyeriana* NC031899.1, *Dipteronia sinensis* MK193777.1, *Aesculus wangii* NC035955.1, Dimocarpus longan MK726005.1, *Sapindus mukorossi* NC025554.1, *Korelreuteria paniculata* NC037176.1, *Dodonaea viscosa* NC036099.1, *Eurycorymbus cavaleriei* NC037443.1, Citrus polytrifolia NC045403.1.

We used RAxML (Stamatakis 2006) with 1,000 bootstraps under the GTRGAMMAI substitution model to reconstruct a maximum likelihood (ML) phylogeny of thirteen published complete plastomes of Sapindaceae, using Citrus *polytrifolia* as outgroups. The phylogenetic analysis indicates that *N. topengii* is closer to *Dimocarpus longan* than other species in this study (Figure 1). Most nodes in the plastome ML trees were strongly supported. With the complete plastome sequence of *N. topengii* plastome now at hand, its resource exploitation and conservation project can be better proceeded, and phylogenetic studies of Sapindaceae can be explored more sufficiently.

## Data Availability

The data that support the findings of this study are openly available in GenBank of NCBI at http://www.ncbi.nlm.nih.gov, reference number MT471264.
